# Taste of Modern Diets: The Impact of Food Processing on Nutrient Sensing and Dietary Energy Intake

**DOI:** 10.1093/jn/nxab318

**Published:** 2021-10-12

**Authors:** Pey Sze Teo, Rachel Tso, Rob M van Dam, Ciarán G Forde

**Affiliations:** Clinical Nutrition Research Centre (CNRC), Singapore Institute of Food and Biotechnology Innovation (SIFBI), Agency for Science, Technology and Research (A*STAR), Singapore; Clinical Nutrition Research Centre (CNRC), Singapore Institute of Food and Biotechnology Innovation (SIFBI), Agency for Science, Technology and Research (A*STAR), Singapore; Saw Swee Hock School of Public Health, National University of Singapore, Singapore; Clinical Nutrition Research Centre (CNRC), Singapore Institute of Food and Biotechnology Innovation (SIFBI), Agency for Science, Technology and Research (A*STAR), Singapore; Department of Physiology, Yong Loo Lin School of Medicine, National University of Singapore, Singapore; Sensory Science and Eating Behavior, Division of Human Nutrition and Health, Wageningen University, Wageningen, The Netherlands

**Keywords:** taste quality and intensity, nutrient content, dietary energy intake, BMI, NOVA food processing classification

## Abstract

**Background:**

Both fresh and processed foods are available in the modern food environment where taste can signal presence of nutrients. However, whether these taste–nutrient relationships are maintained across different degrees of food processing is not well understood, and less is known about the relative contribution of different taste qualities to population energy intakes.

**Objectives:**

To investigate the association between perceived intensity of 6 taste modalities and a food's nutrient content in the context of food processing and to further examine the relative contribution of different taste clusters to total energy intakes, stratified by weight status.

**Methods:**

Diet and lifestyle data from the Singapore Multi-Ethnic Cohort Phase 2 (*N* = 7011; aged 21–75 y) were collected through interviewer-administrated questionnaires. Taste and nutrient profiles for each of the 269 Singaporean foods were derived using a published taste database and food composition table. Each food was then categorized into the NOVA food-processing classification (unprocessed, processed, ultra-processed) to compare the strength of taste–nutrient relationships. Multivariable-adjusted models were used to examine associations between relative consumption of foods from different taste clusters and processing categories, energy intake, and BMI (in kg/m^2^) within a population cohort.

**Results:**

Sweet taste and mono- and disaccharide content of foods were significantly associated across all processing categories, although this association was weaker among ultra-processed foods (UPFs) (*r* = 0.42) than among unprocessed foods (*r* = 0.72). In contrast, associations between fat sensation and fat content (*r* = 0.74), as well as salt taste and sodium content (*r* = 0.84), were stronger for UPFs. Individuals who had higher energy intakes or were overweight (BMI >23) derived significantly greater percentage of energy from processed foods rather than UPFs, and this energy was higher from “savory–fatty” and lower from “neutral” tasting foods than those with lower energy intakes and normal weight (all *P* < 0.001). Eighty percent of individuals’ dietary energy was from both “savory–fatty” and “neutral” foods, independent of differences in total energy intake and weight status.

**Conclusions:**

Taste–nutrient relationships are maintained across different degrees of food processing. Greater consumption of foods that have a high “savory–fatty” taste was associated with increased energy intakes and overweight in the Asian population.

## Introduction

Taste quality and intensity play an important role in food choice and energy intake, and a food's taste is thought to signal its nutrient density (1). Sweet taste signals the presence of simple sugars, glutamate salts and inosine stimulate umami taste and are associated with protein, and salt taste signals the presence of sodium and other electrolytes (2). Bitter taste is often associated with alkaloids and glycoside compounds, whereas sour taste is associated with the presence of organic acids commonly found in fruits (3, 4). These taste–nutrient relationships help to guide our food choices (5) and serve to counteract dietary imbalances (6, 7) by indicating a food's safety, ripeness, and energy content (8–10). The current food environment is characterized by both fresh and processed foods with different degrees of processing (11). Whether a food's taste quality and intensity still relate to the macronutrient content of modern highly processed foods remains poorly understood.

Concerns have been raised about the consumption of highly processed foods and their contribution to higher sugar, saturated fat, salt, and energy intakes (11–14). In recent years, there has been an increased application of taste active ingredients and additives to maintain an optimal sensory appeal of reformulated products while reducing their salt, sugar, and saturated fat content (15, 16). Wide variations in fat, sugar, and salt content of highly processed and reformulated foods could weaken the link between a food's taste quality and intensity, as well as its nutrient content. Similarly, the ability to discriminate the fat and energy content of foods decreases at higher taste intensities, as demonstrated for sweet and salty tastes (17, 18). Modern food formulation also makes it possible to retain a high taste intensity without the associated nutrient impact and may disrupt associations between a food's nutritive density and its predominant taste quality and intensity. For example, it is possible to have the sweet taste quality and intensity associated with high amounts of free sugars while reducing or removing sugars and replacing them with low- or no-calorie sweeteners (19). In addition, the presence of 2 or more tastants in the ultra-processed foods may affect taste perception via taste–taste interactions, reducing the clarity of the taste signal in mixed dishes (20). Salt is often added to formulated foods to suppress bitter tastes and, through this, enhance the sweetness perception by releasing sugars from antagonistic mixture suppressions (21). Similarly, savory enhancers such as monosodium glutamate and inosine monophosphate can produce a strong umami or savory taste, in the absence of protein (22). The high sodium content of gingerbread is not reflected as a perceived “salty” taste due to suppression by its high sweetness/sugar content (23). Even staple foods such as bread are often perceived as neutral in taste but can contain up to 3 g sugar per slice, suggesting the potential for a disconnect between a food's nutrient content and its perceived taste quality and intensity.

If food formulation were to disrupt the relation between a food's taste intensity and nutrient signaling (24), it could result in a reduced perceptual salience for sensory cues and decrease our ability to detect and adjust intakes in response to the foods being consumed. Covert manipulation of a food's energy density can lead to acute overconsumption of energy within a meal, and research has shown we tend to poorly compensate for these calorie deviations at later meals (25–27). A recent inpatient randomized controlled trial showed that extended consumption of a highly processed diet resulted in sustained higher daily energy intake and increased body weight and adiposity compared with consuming a less-processed diet (28). These findings have led to speculation on the potential mechanisms for increased energy intake from a diet high in processed foods and how food formulation and processing may influence taste perception and detection of nutrient density.

Five studies to date have explored taste–nutrient associations across the food supplies of the Netherlands (29, 30), Australia (31), Malaysia (30), France (32), and the United States (33). These studies show strong positive relations between sweetness and mono- and disaccharides, saltiness and sodium, umami/savory taste and protein, and fat sensation and fat content. The energy content of a food was also found to be positively associated with fat sensation but not sweetness intensity in the American, Australian, and Malaysian diets (30, 31, 33). Previous research suggests that taste–nutrient associations tend to be more pronounced in raw and moderately processed foods than in highly processed foods (29). This creates the possibility that the taste quality of processed foods may not be reflected in their nutrient contents and could contribute to nutritional imbalance or decrease the relevance of sensory cues in directing our consumption behavior. A recent French study showed that certain taste–nutrient associations were weaker for ultra-processed foods compared with the same relations in minimally or unprocessed foods (32). However, whether these differences apply to food consumption patterns in other populations with different culinary traditions remains unknown, and the contribution of different taste qualities to population dietary energy intakes remains unclear.

The current study examined taste–nutrient relationships across a representative set of foods from the Singaporean diet across unprocessed, processed, and ultra-processed categories as defined by the NOVA classification system (34). In a second step, we examined the contribution of foods from different taste clusters to total energy intake (i.e., by food weight and percentage of energy) and compared this by weight status population subgroups across diets that differ in their degree of food processing (i.e., unprocessed, processed, and ultra-processed).

## Methods

### Study overview

Commonly consumed Singaporean foods (*N* = 263) were selected from a validated FFQ (35) and classified into unprocessed, processed, or ultra-processed food categories based on their degree of processing as defined by the NOVA classification system (34). The predominant taste profile for each food was taken from a published taste database (30) and associated with the nutrient content of each food as derived from nutrient composition tables. For the comparison of energy intakes by taste quality, the relative contribution of each taste cluster to total energy intake was assessed across quartiles of dietary energy intake (i.e., from low to very high) within a cross-sectional population survey (*N* = 7011). The differences of intakes from taste clusters were also further stratified by weight status of cohort participants.

### Study population

We used data from the follow-up Singapore Multi-Ethnic Cohort Phase 2 (MEC2) study 2016–2019, which comprised Singaporean citizens and permanent residents aged 21–75 y. This study population includes 3 major Asian ethnic groups: Chinese, Indian, and Malay. Detailed information on the MEC2 cohort is published elsewhere (36) and can be found at http://blog.nus.edu.sg/sphs/the-first-sphs-follow-up/. The present study included individuals who participated in both interview and health screening sessions (*n* = 7314) and excluded those with missing data (*n* = 86), invalid energy intake (i.e., extreme energy intakes of ≤500 kcal/d or ≥6000 kcal/d) (*n* = 139), and those with major chronic diseases (i.e., cancer, heart attack, or stroke; *n* = 78). A total of 7011 participants were included in the final analysis. Written informed consent was obtained from all participants, and the study protocol was approved by the Institutional Review Board of the National University of Singapore (NUS-IRB B-16–125).

### Assessment of energy consumption and dietary intake data

The FFQ used in this study was previously developed using nationally representative data to cover 95% of daily energy intake in a Singaporean population and accounting for 89% of between-person variation in energy intakes (37). The Singaporean foods listed were recorded using a validated semiquantitative FFQ (35), comprising 163 food items, with additional subquestions on food subtypes (e.g., types of noodles/rice used), associated ingredients (e.g., added oil and sugar), and cooking methods (i.e., curries with or without coconut, stir-fried, deep-fried, stewed, roasted, and boiled). The food subtypes, associated ingredients, and specified cooking methods in the FFQ were further converted into whole dishes as consumed. For example, “Fresh chicken” and “In curry with coconut” was recoded as “Chicken; curry with coconut.” Beverages with extra added sugar were recoded as new food items in combination with sugar. This resulted in an extended list of 269 commonly consumed foods and beverages.

### Defining foods’ predominant taste profiles using the “food taste” database

The predominant sensory taste profile for the 269 Singaporean foods in the FFQ was drawn from a published taste database of 892 foods and beverages (30). This standardized database of food taste qualities was developed using a trained sensory panel to objectively profile the intensity of 5 basic tastes (i.e., sweet, sour, bitter, umami, salt) and fat sensation across a large and representative set of foods and beverages using a 100-point rating system based on the Spectrum method (38). The taste quality is with reference to the 6 taste modalities profiled (i.e., sweet, sour, bitter, umami, salt, and fat sensation), whereas taste intensity is the quantified taste scores referring to the strength or weakness of the taste qualities. For untested foods/mixed dishes not covered in the published food taste database, taste profiles were matched and imputed using similar foods to those tested within the same food group, taking account of the foods’ nutrient and energy content and cooking method. For example, taste profiles of untested blueberries were imputed using the tested strawberries from the same food group due to their similar nutrient and energy content. The 269 foods included in the current study represented >95% of the food sources of energy intake in Singapore, and each food was assigned a predominant taste quality and intensity from 5 dietary taste clusters: “sweet–fatty,” “savory–fatty,” “sweet–sour,” “neutral,” and “bitter” (30), in line with previous approaches (30, 39).

### Classification of food items into unprocessed, processed, and ultra-processed categories

The 269 foods were classified to 1 of the 3 groups based on their degree of food processing from unprocessed foods to processed foods, ultra-processed foods (UPFs), and culinary ingredients using the NOVA classification (34). Unprocessed or minimally processed foods are natural foods that had been subjected to minimal or no processing. Culinary ingredients include sugar, animal fat (butter), vegetable oils, starches, salt, and vinegar. Processed foods are defined as combining culinary ingredients (fats, sugar, salt) with unprocessed/fresh foods and are generally consumed as part of meals or dishes. UPFs are described as industrial creations that contain ingredients not found in home cooking, in addition to fats, sugar, and salt (34). Culinary ingredients are not consumed in isolation but rather in combination with processed or UPFs and were removed from further analysis, leaving a final set of 267 foods. As the NOVA classification system is open to interpretation and relies on available food ingredient and processing information, 3 researchers independently grouped the foods into unprocessed, processed, and UPFs, and any discordance in classification was discussed and carefully considered to reach a consensus, in line with previous approaches (40, 41). The final list of food items and their processing category are summarized in **Supplemental Table 1**.

### Nutrient contents of selected Singaporean foods

The nutrient values for each food item were collated from a food composition database from the Singapore Health Promotion Board (42) and USDA National Nutrient Database for Standard Reference (43). Recipe calculation was used to estimate the nutrient composition for complex mixed dishes and prepared beverages with added milk and/or sugars. For the comparison across processing categories, the nutrients of interest included energy (kcal), protein (g), fat (g), carbohydrates (g), mono- and disaccharides (g), dietary fiber (g), and sodium (mg), and all nutrient values are given per 100 g of the edible parts of each food.

### Assessment of body composition and covariates

Details on the assessment procedures for body composition are reported in our previous study (44). In brief, body weight and height were assessed according to WHO standards and were taken to the nearest 0.1 kg  or 0.1 m, respectively, by trained personnel. BMI was calculated by dividing weight (kg) by height squared (m^2^). Asian cutoffs for BMI classification of overweight (≥23) were used to identify individuals at moderate risk of obesity-related diseases.

Data on sociodemographic characteristics, medical history, and dietary and other lifestyle factors were recorded from all participants through face-to-face interviews by trained staff. Physical activity was assessed using a locally validated SP2 Physical Activity Questionnaire (45), which assessed activity in the leisure, occupational, and transport domain. Total physical activity was expressed in metabolic equivalent task units (METs) based on the Ainsworth compendium (46).

### Statistical analysis

#### At food level

Four extreme food products (jam, margarine, dried fish, and preserved vegetables) were excluded from the final data set, as they had taste values >3 SDs from the mean, resulting in a total of 263 Singaporean foods in the final model. Descriptive statistics were reported as mean ± SD, unless otherwise indicated. Analysis of variance (ANOVA) was used to determine the differences of the taste intensity and nutrient content across foods from 3 categories of processing (i.e., unprocessed, processed, and ultra-processed). Pearson correlations were calculated between sweet, sour, bitter, umami, salt, and fat sensation and the respective macronutrient content of each food. The correlation analysis model was repeated without beverages. Differences in taste–nutrient associations across processing categories were compared based on overlap of 95% CIs of Pearson correlation coefficients that were calculated using the Fisher *z*′ transformation. Simple linear regression was performed between taste intensity ratings and nutrient content for the unprocessed, processed, and ultra-processed foods.

#### At population level

Quartiles were created both for the proportion of energy intake (percentage of energy) from processed and ultra-processed foods and for the dietary energy intake (kcal/d) of participants. A Pearson χ^2^ test was used to evaluate the differences of the categorical variables across the quartiles of processed and ultra-processed food intake, whereas ANOVA was used to determine differences for all continuous variables across the processed and ultra-processed food intake groups. The contributions of amount consumed (g and percentage of energy) from foods in different taste clusters to quartiles of dietary energy intake (i.e., from low to very high) were further analyzed after adjusting for all known potential confounders, including age (y), sex (male or female), ethnicity (Chinese, Malay, Indian), education level (primary or below, secondary, higher education including vocational, university), total physical activity (MET-min/wk), BMI, smoking (yes or no), and alcohol drinking status (yes or no). A multivariable-adjusted ANCOVA model was used to test differences in amount consumed (g and percentage of energy) from taste clusters by weight status, considering all taste clusters simultaneously. The relative contributions of energy intake from foods that differed in processing categories across quartiles of dietary energy intake were also assessed after multivariable adjustment.

All statistical analysis was performed using IBM SPSS Statistics (version 26.0; IBM Corp.), and a *P* < 0.05 was considered significant.

## Results

### Taste segments across processing categories

**Table 1** shows the mean taste intensity and nutrient content of commonly consumed Singaporean foods grouped by food processing categories (i.e., unprocessed, processed, and ultra-processed). Of the 263 total foods, 28% were categorized as unprocessed foods, 31% as processed foods, and 41% as UPFs. Significant differences were observed in perceived intensity for umami, salt, and sweet tastes and fat sensation across different categories of processing. Unprocessed foods were rated as lower in intensity across all taste qualities (all below 15 points), with “neutral” taste being the most dominant. Processed foods were significantly higher in intensity for umami, salt, and fat taste (i.e., 22–31 points) compared with both unprocessed foods and UPFs (all *P* < 0.001). The UPFs were significantly higher in “fat” (21 points) and “sweet” (28 points) taste intensities compared with unprocessed foods. Nutrient contents also differed across processing categories, with processed foods and UPFs having significantly higher energy density, fat, and sodium content and lower dietary fiber content than unprocessed foods. UPFs also had significantly lower protein and sodium content and were higher in carbohydrates and mono- and disaccharides compared with processed foods (all *P* < 0.001).

**TABLE 1:** Mean taste intensity and nutrient contents of 263 Singaporean foods by their NOVA classification

										*P* value		
	Unprocessed foods (*n* = 72)			Processed foods (*n* = 82)			Ultra-processed foods (*n* = 109)			Unprocessed vs.	Unprocessed vs.	Processed vs.
Characteristic	Mean	SD	Range	Mean	SD	Range	Mean	SD	Range	processed	ultra-processed	ultra-processed
Taste intensity, score (range: 0–100)												
Sweet	12.8	11.2	0–45.7	8.70	9.80	0–60.2	27.7	16.3	0–65.7	0.054	<0.001	<0.001
Sour	9.80	14.8	0–71.0	4.60	5.90	0–27.7	5.50	10.2	0–67.0	0.003	0.009	0.586
Bitter	4.60	8.90	0–63.0	5.70	13.2	0–60.0	9.60	14.5	0–60.0	0.594	0.012	0.041
Umami	7.90	11.8	0–47.0	21.6	11.8	0–42.7	4.30	7.30	0–38.6	<0.001	0.020	<0.001
Salt	7.90	11.6	0–53.0	27.2	14.0	0–65.0	8.30	10.8	0–40.0	<0.001	0.828	<0.001
Fat sensation	13.3	11.6	0–57.0	31.0	13.1	0–67.0	20.7	15.7	0–69.0	<0.001	0.001	<0.001
Nutrient, unit/100 g												
Energy, kcal	79.3	95.4	0–574	147	82.9	0–347	145	145	0–603	<0.001	<0.001	0.887
Protein, g	4.10	5.90	0–28.0	9.30	8.20	0–32.4	3.70	4.50	0–26.1	<0.001	0.664	<0.001
Fat, g	2.70	8.70	0–52.5	7.90	7.60	0–35.0	5.70	8.70	0–48.4	<0.001	0.022	0.071
Carbohydrates, g	10.0	10.0	0–63.9	9.00	10.4	0–46.7	20.4	20.3	0–74.2	0.685	<0.001	<0.001
Mono- and disaccharides, g	4.10	5.90	0–38.1	1.80	2.80	0–16.6	10.3	10.5	0–57.2	0.061	<0.001	<0.001
Dietary fiber, g	2.10	2.30	0–10.8	1.20	1.50	0–8.10	1.20	1.60	0–6.60	0.002	0.001	0.953
Sodium, mg	77.2	139	0–57.0	301	257	0–1280	164	253	0–1200	<0.001	0.013	<0.001

**FIGURE 1:**
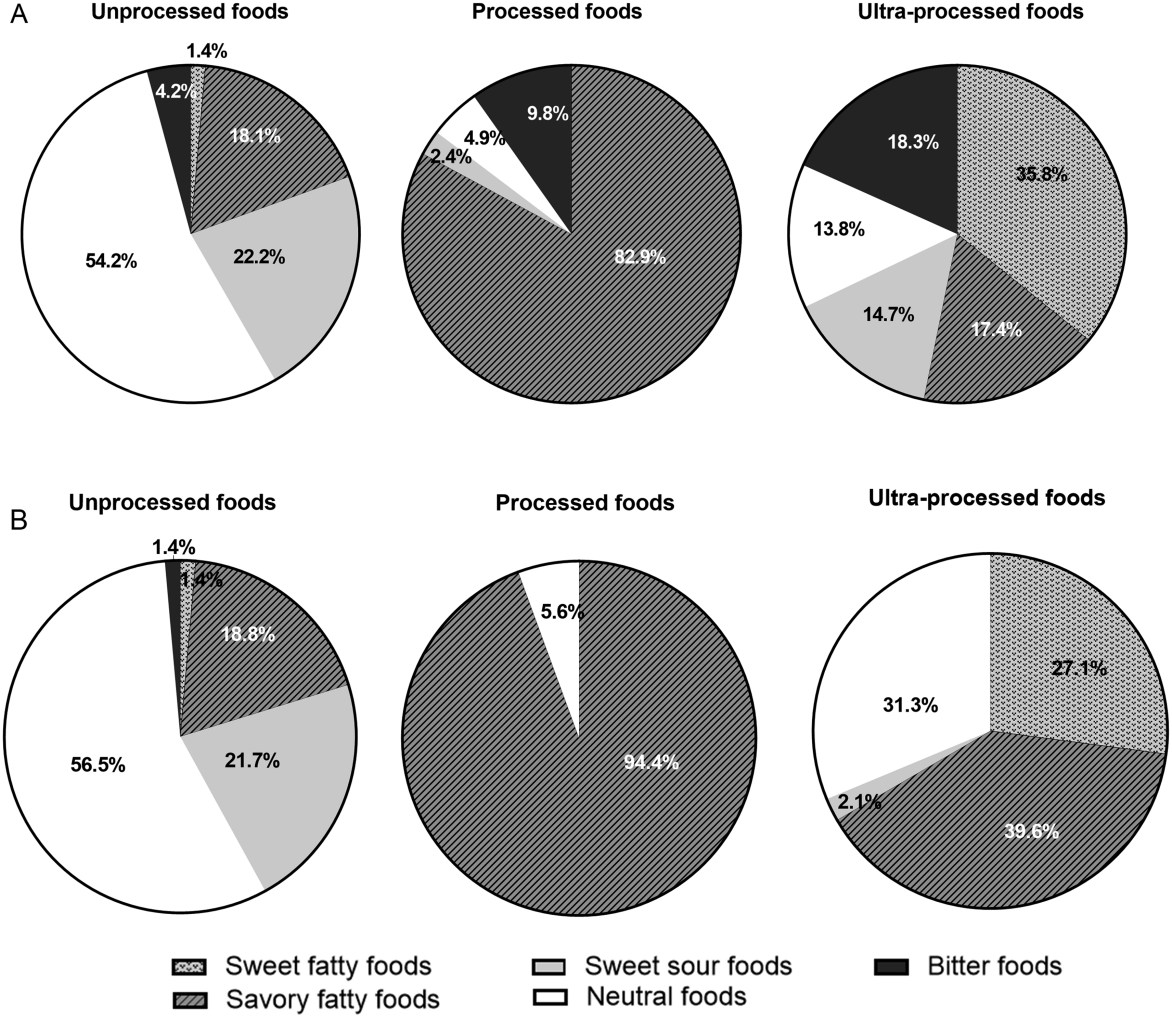
Proportion of taste clusters of commonly consumed foods in Singapore by degree of food processing (NOVA classification: unprocessed, processed, and ultra-processed). Foods and beverages (*n* = 263) (A) and foods only (*n* = 189) (B).

The split between taste cluster segments for each processing category is illustrated in **Figure 1**A for all foods and beverages and in Figure 1B for foods only. The unprocessed category (54%) compiled mainly foods with a “neutral” taste, whereas processed foods (83%) were dominated by mixed dishes high in “savory–fatty” taste. The UPF category was dominated by sweet–fatty (36%), savory–fatty (17%), and bitter foods (18%). When beverages were removed and taste clusters were compared across processing levels (Figure 1B), unprocessed taste segments were unaffected, but the bitter taste cluster disappeared from both processed and UPF categories, confirming the bitter taste segment was mainly driven by bitter tasting beverages, such as coffee, tea, and beer.

### Taste–nutrient relationships across processing categories

**Table 2**, **Supplemental Table 2**, and **Figure 2** summarize the associations between taste quality and intensity and its nutrient content for unprocessed, processed, and ultra-processed foods. Sweetness intensity was positively correlated with a food's mono- and disaccharide content across all processing categories (all *P* < 0.01) and with carbohydrate content for unprocessed foods (*r* = 0.30, *P* = 0.01) but not processed or ultra-processed foods. Sweet taste intensity was not substantially correlated with the energy and fat content of foods but was positively correlated with energy and fat content for UPFs, after beverages were removed from the analysis (Table 2). Sweet taste intensity was explained by mono- and disaccharide content, and this was strongly observed in unprocessed foods (*R*^2^ = 0.51, *P* < 0.001), followed by the processed (*R*^2^ = 0.24, *P* < 0.001) and ultra-processed (*R*^2^ = 0.18, *P* < 0.001) categories.

**FIGURE 2:**
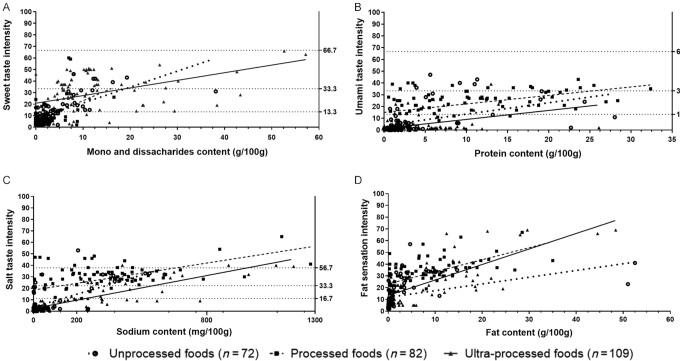
The associations between sweet taste and mono- and disaccharides (A), between umami and protein (B), between salt taste and sodium (C), and between fat sensation and fat (D) in unprocessed, processed, and ultra-processed foods and beverages (*n* = 263), as defined by the NOVA classification.

**TABLE 2:** Pearson correlation between taste intensity and nutrients across unprocessed, processed, and ultra-processed food items (*N* = 189)^1^

Characteristic	Energy	Protein	Fat	Carbohydrates	Mono- and disaccharides	Dietary fiber	Sodium
Model: Foods only							
Unprocessed foods (*n* = 69)							
Sweet	−0.02	−0.20	−0.11	0.31**	0.73^**b^	0.19	−0.16
Sour	−0.10	−0.14	−0.09	0.01	0.32^**a^	−0.05	−0.05
Bitter	−0.05	0.12	0.00	−0.15	−0.16	0.16	−0.10
Umami	0.13	0.47**	0.11	−0.19	−0.27*	−0.29*	0.76^**ab^
Salt	0.32**	0.58^**b^	0.28*	−0.10	−0.28*	−0.05	0.67**
Fat sensation	0.45**	0.58^**a^	0.43**	−0.05	−0.15	0.02	0.54**
Processed foods (*n* = 72)							
Sweet	0.04	0.10	−0.02	0.05	0.39**	0.00	0.11^a^
Sour	−0.04	−0.04	−0.02	−0.04	0.09	0.03	0.20
Bitter	−0.12	−0.07	0.01	−0.11	0.08	0.01	−0.07
Umami	−0.08	0.34**	0.02	−0.35**	0.02^a^	−0.23	0.34**
Salt	0.17	0.08	0.36**	−0.26*	−0.08	−0.18	0.39**
Fat sensation	0.44**	0.26*	0.54**	−0.18	0.07	−0.11	0.44**
Ultra-processed foods (*n* = 48)							
Sweet	0.35*	−0.16	0.32*	0.18	0.68**	−0.03	−0.49**
Sour	−0.22	−0.20	0.12	−0.37*	−0.20	−0.36*	0.40**
Bitter	0.28	−0.01	0.34*	0.06	0.33*	0.04	−0.03
Umami	−0.36*	0.25*	−0.01	−0.49**	−0.49**	−0.15	0.41**
Salt	−0.07	0.22	0.33*	−0.38**	−0.48**	−0.08	0.71**
Fat sensation	0.19	0.10	0.69**	−0.42**	0.05	−0.27	0.33*
All foods combined (*N* = 189)							
Sweet	0.29**	−0.16*	0.14	0.39**	0.73**	0.16*	−0.18*
Sour	−0.17*	−0.17*	−0.06	−0.14	0.22	−0.08	0.06
Bitter	−0.14	−0.05	−0.05	−0.13	−0.03	0.11	−0.17*
Umami	−0.05	0.48**	0.11	−0.38**	−0.39**	−0.33**	0.48**
Salt	0.22**	0.44**	0.38**	−0.21**	−0.31**	−0.20**	0.62**
Fat sensation	0.44**	0.42**	0.61**	−0.11	0.01	−0.17*	0.55**

1 Correlation (2-tailed) significant at *P < 0.05 and **P < 0.01. Different from ^a^ultra-processed and ^b^processed foods based on non-overlapping 95% CIs.

Umami taste was positively correlated with a food's protein and sodium content across all processing categories. For unprocessed foods, umami was more strongly associated with sodium (*r* = 0.76, *P* < 0.001) than with protein content (*r* = 0.48, *P* < 0.001), whereas the strength of associations between umami and its sodium and protein content was similar for processed and ultra-processed foods. A food's energy content was only associated with its umami taste intensity in the processed food category. A food's umami taste intensity was similarly explained by its protein content for unprocessed (*R*^2^ = 0.23, *P* < 0.001), processed (*R*^2^ = 0.24, *P* < 0.001), and ultra-processed (*R*^2^ = 0.22, *P* < 0.001) food categories.

Salt taste intensity was positively associated with a food's sodium content in unprocessed (*R*^2^ = 0.45, *P* < 0.001), processed (*R*^2^ = 0.29, *P* < 0.001), and ultra-processed (*R*^2^ = 0.70, *P* < 0.001) foods.

Fat sensation was positively correlated with a food's protein, energy, and sodium content across all processing categories. Fat content substantially explained perceived fat sensation in unprocessed (*R*^2^ = 0.18 *P* < 0.001), processed (*R*^2^ = 0.35 *P* < 0.001), and ultra-processed (*R*^2^ = 0.54, *P* < 0.001) foods.

Bitter taste was significantly inversely correlated with energy content and most of the other nutrients in the processed and ultra-processed foods but not among unprocessed foods.

The strength of the associations between taste intensity and nutrient contents differed significantly across foods from different processing categories. The association between sweet taste and mono- and disaccharides was stronger among unprocessed foods (*r* = 0.72; 95% CI: 0.59, 0.82) than among ultra-processed foods (*r* = 0.42; 95% CI: 0.25, 0.56). In contrast, fat sensation had a weaker correlation with the fat content of foods among unprocessed food (*r* = 0.43; 95% CI: 0.22, 0.60) than among ultra-processed food (*r* = 0.74; 95% CI: 0.64, 0.81). The strength of these associations remained unaffected after beverages were removed from the model.

### Associations between taste clusters, processed foods, energy intakes, and weight status

**Table 3** summarizes sociodemographic and lifestyle characteristics (*N* = 7011) of participants across quartiles of processed and ultra-processed food intakes as a percentage of total energy intake. The largest ethnic group were Chinese (71.1%), followed by Indians (14.7%), Malays (9.0%), and others (5.2%), broadly in line with population ethnic distribution for Singapore. Higher intakes of processed and ultra-processed foods were associated with being male, younger, and of Malay ethnicity, as well as having higher body weight, BMI, and physical activity levels than those with lower intakes of processed and ultra-processed foods (all *P* < 0.001). Individuals in the highest quartile of processed and ultra-processed food intake had significantly higher intake of total energy, macronutrients, percentage of energy from macronutrients, total amount of foods (g), and foods from all 5 taste clusters (g). However, those with the highest processed and ultra-processed intakes derived a greater proportion of their daily energy from sweet–fatty, savory–fatty, and bitter tasting foods, with smaller relative intakes of neutral and sweet–sour foods, compared with those with lower intakes (all *P* < 0.001).

**TABLE 3:** Characteristics of participants according to quartiles of processed and ultra-processed food intake in the Singapore Multi-Ethnic Cohort 2 (*N* = 7011)^1^

	Quartiles of processed and ultra-processed food intake				
Characteristic	Low: 51.8% (*n* = 1752)	Medium: 67.4% (*n* = 1753)	High: 76.0% (*n* = 1753)	Very high: 85.9% (*n* = 1753)	*P-*trend
Age, y	54.1 ± 12.4^a^	50.7 ± 12.8^b^	47.6 ± 12.8^c^	46.9 ± 12.6^c^	<0.001
Sex, %					
Men	21.8	24.8	24.7	28.7	<0.001
Women	27.5	25.2	25.2	22.0	
Ethnic group, %					
Chinese	27.2	25.4	24.5	22.9	<0.001
Malay	19.3	21.7	27.4	31.6	
Indian	20.1	26.9	25.3	27.7	
Others	18.2	20.1	27.5	34.2	
Height, m	1.61 ± 0.09^d^	1.62 ± 0.09^c^	1.63 ± 0.09^b^	1.64 ± 0.09^a^	<0.001
Body weight, kg	63.1 ± 13.1^d^	65.0 ± 13.2^c^	67.0 ± 14.8^b^	68.5 ± 14.8^a^	<0.001
BMI, kg/m^2^	24.3 ± 4.49^c^	24.6 ± 4.36^c^	25.1 ± 4.78^b^	25.4 ± 4.85^a^	<0.001
Dietary energy intake, kcal/d	1900 ± 799^d^	2270 ± 869^c^	2540 ± 972^b^	2760 ± 1150^a^	<0.001
Protein intake, g/d	74.1 ± 36.1^d^	89.3 ± 39.8^c^	99.4 ± 43.0^b^	104 ± 47.7^a^	<0.001
Fat intake, g/d	70.7 ± 40.2^d^	89.5 ± 40.7^c^	104 ± 45.9^b^	115 ± 53.4^a^	<0.001
Carbohydrate intake, g/d	241 ± 100^d^	276 ± 104^c^	301 ± 114^b^	325 ± 136^a^	<0.001
Sugar intake, g/d	64.1 ± 34.8^d^	77.4 ± 36.5^c^	86.5 ± 39.3^b^	102.7 ± 50.4^a^	<0.001
Dietary fiber intake, g/d	18.9 ± 9.59^c^	20.2 ± 8.79^b^	21.3 ± 8.56^a^	21.6 ± 9.74^a^	<0.001
Sodium intake, mg/d	2510 ± 1270^d^	3230 ± 1430^c^	3780 ± 1630^b^	4140 ± 1950^a^	<0.001
Total amount consumed, g/d	1980 ± 779^d^	2230 ± 820^c^	2370 ± 865^b^	2500 ± 1010^a^	<0.001
Amount consumed, g/d					
Sweet–fatty foods	99.0 ± 116^d^	148 ± 149^c^	174 ± 161^b^	218 ± 203^a^	<0.001
Savory–fatty foods	598 ± 325^d^	815 ± 376^c^	982 ± 448^b^	1090 ± 566^a^	<0.001
Sweet–sour foods	246 ± 215^c^	267 ± 217^b^	280 ± 218^b^	312 ± 305^a^	<0.001
Neutral foods	806 ± 385^a^	713 ± 317^b^	630 ± 261^c^	503 ± 286^d^	<0.001
Bitter foods	232 ± 218^d^	286 ± 241^c^	305 ± 274^b^	376 ± 316^a^	<0.001
Amount consumed, % energy					
Sweet–fatty foods	4.82 ± 4.66^d^	6.65 ± 4.99^c^	7.75 ± 5.48^b^	9.12 ± 6.50^a^	<0.001
Savory–fatty foods	44.9 ± 13.0^d^	52.3 ± 10.8^c^	56.9 ± 10.7^b^	59.2 ± 12.4^a^	<0.001
Sweet–sour foods	7.93 ± 5.69^a^	6.63 ± 4.35^b^	5.94 ± 3.73^c^	6.06 ± 4.14^c^	<0.001
Neutral foods	39.7 ± 13.0^a^	31.3 ± 9.49^b^	26.1 ± 8.40^c^	21.0 ± 8.95^d^	<0.001
Bitter foods	2.70 ± 3.19^c^	3.14 ± 3.40^b^	3.27 ± 3.81^b^	4.59 ± 5.14^a^	<0.001
Total physical activity, MET-min/wk	1110 ± 1030^c^	1140 ± 986^c^	1180 ± 1010^b^	1290 ± 1110^a^	<0.001

1 Unadjusted data. Labeled means in a row without a common letter differ at *P* < 0.05. Values are presented as mean ± SD unless otherwise indicated. MET, metabolic equivalent task units.

To examine the associations of consuming different proportions of foods from each processing category and dietary energy intake, we compared the relative contribution of each processing category across low, medium, high, and very high energy intake quartiles (**Table 4**). Higher dietary energy intake was significantly associated with consumption of more energy from processed and ultra-processed foods and lower intakes of unprocessed foods. These differences were subtle, with those in the “very high” energy intake quartile consuming only 3% more of their daily energy from ultra-processed foods compared with those in the lowest energy intake quartile, despite a 3-fold difference in average daily energy intakes between the 2 quartiles. These associations remained significant after adjustment for potential confounders. Individuals who were overweight consumed significantly more energy from processed foods and less energy from unprocessed foods compared with those in the normal-weight range (all *P* < 0.001). No association was observed between ultra-processed food consumption and being overweight (*P* = 0.815) following multivariable adjustment (data not shown).

**TABLE 4:** Amount (percentage of energy) consumed as foods with different degrees of food processing: unprocessed, processed, and ultra-processed across quartiles of total energy intake (EI) of cohort participants (*N =* 7011)^1^

	% energy, mean ± SEM			
Characteristic	Low EI (quartile 1; *n* = 1752)	Medium EI (quartile 2; *n* = 1753)	High EI (quartile 3; *n* = 1753)	Very high EI (quartile 4; *n* = 1753)
Unprocessed	36.3 ± 0.30	33.5 ± 0.29	30.0 ± 0.29	26.0 ± 0.29
Processed	38.0 ± 0.30	40.4 ± 0.29	42.4 ± 0.29	44.4 ± 0.29
Ultra-processed	27.4 ± 0.31	27.0 ± 0.30	29.0 ± 0.30	30.4 ± 0.30

1 Estimates were adjusted for age (y), sex (male or female), ethnicity (Chinese, Malay, Indian), education level (primary or below, secondary, higher education including vocational, university), total physical activity (metabolic equivalent task units–/wk), BMI, smoking (yes or no), and alcohol consumption status (yes or no). Percentage of energy consumed from different processing categories differed significantly across quartiles of total energy intake (all *P*-trend < 0.001).

**FIGURE 3:**
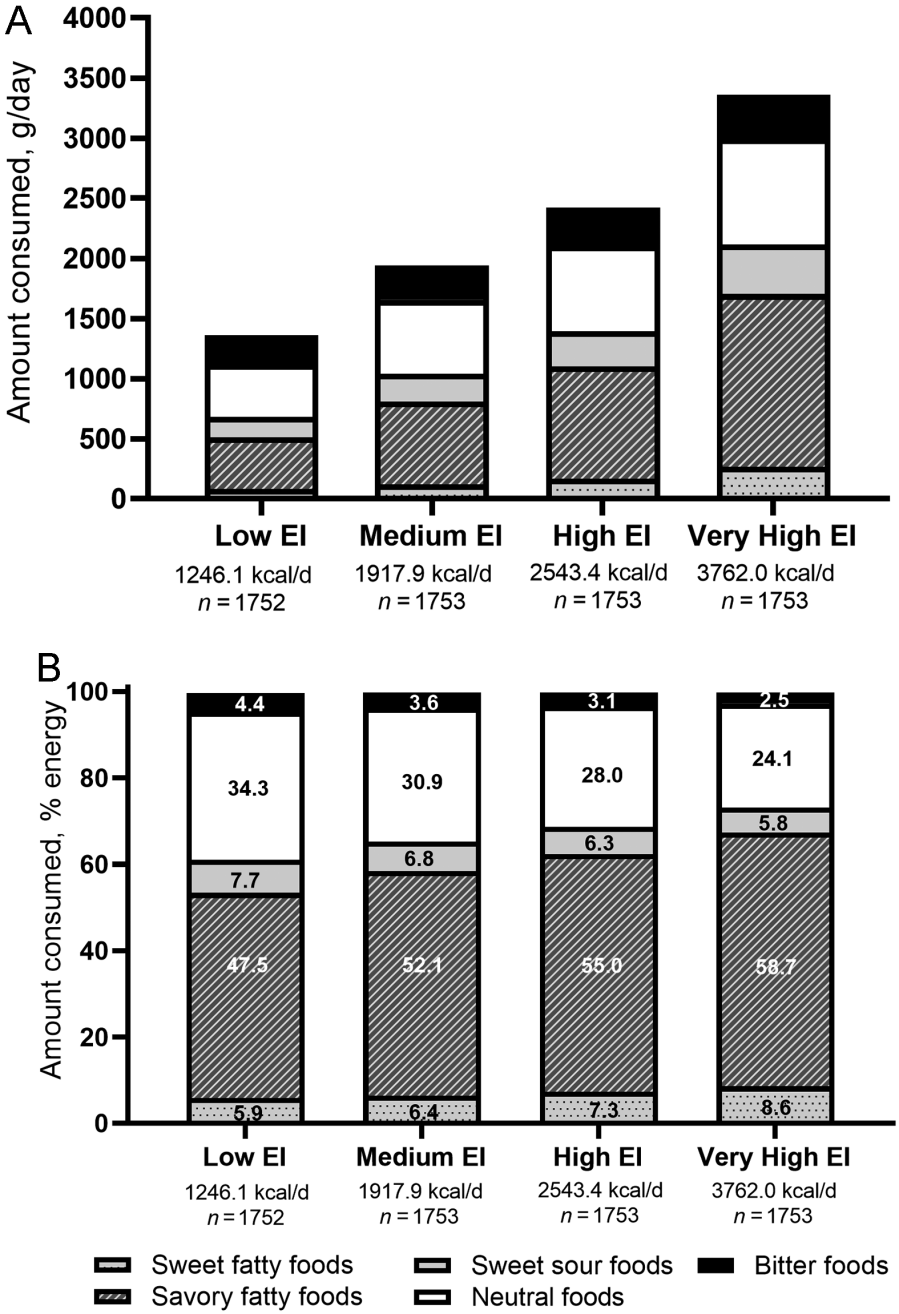
The amount (g/d) (A) and percentage of energy (B) consumed as foods with different taste clusters (sweet–fatty, savory–fatty, sweet–sour, neutral, and bitter) according to quartiles of total energy intake of cohort participants (*N*_total_ = 7011). Estimates were adjusted for age (y), sex (male or female), ethnicity (Chinese, Malay, Indian), education level (primary or below, secondary, higher education including vocational, university), total physical activity (metabolic equivalent task units–min/wk), BMI, smoking (yes or no), and alcohol consumption status (yes or no). The amount (g/d) and percentage of energy consumed from different taste clusters differed significantly across quartiles of total energy intake (all *P*-trend < 0.001). EI, energy intake.

**Figure 3** shows the amount consumed (g) and the relative contribution (%) from each taste cluster to daily energy intake across energy intake quartiles after multivariable adjustment. As total energy intake increased from low to very high, the amount of foods consumed across all 5 taste clusters (g) also significantly increased (all *P*-trend < 0.001). However, lower daily energy intakes were associated with a higher proportion of energy coming from foods in neutral, sweet–sour, and bitter taste clusters, whereas high and very high daily energy intakes were associated with higher intake of savory–fatty and sweet–fatty tasting foods. Those in the highest energy intake quartile consumed ∼60% of their energy from savory–fatty foods, which was 35% higher than energy consumed from neutral tasting foods (24%). By contrast, those in the lowest quartile of energy intake consumed one-third of their calories from the neutral tasting foods, which was 10% higher compared with those in the highest energy intake quartile. Despite large differences in energy intake from the lowest to highest quartile, the proportion of energy within each quartile was similar from savory–fatty and neutral foods, which represented ∼80% of daily energy intakes, and this taste pattern was maintained across all energy intake groups from low to very high (Figure 3).

**FIGURE 4:**
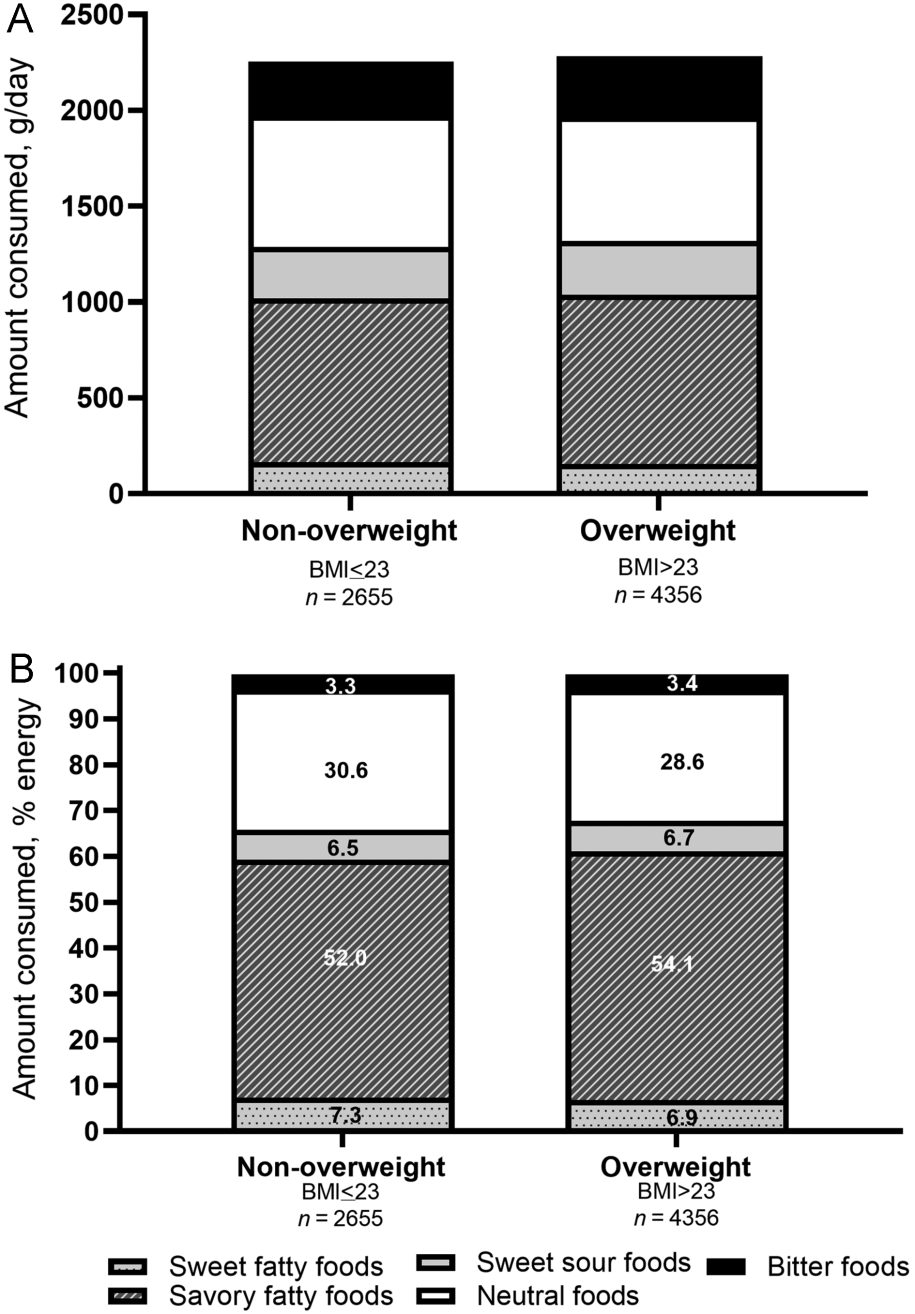
The amount (g/d) (A) and percentage of energy (B) consumed as foods with different taste clusters (sweet–fatty, savory–fatty, sweet–sour, neutral, and bitter) according to weight status of cohort participants (*N*_total_ = 7011). Estimates were adjusted for age (y), sex (male or female), ethnicity (Chinese, Malay, Indian), education level (primary or below, secondary, higher education including vocational, university), total physical activity (metabolic equivalent task units–min/wk), smoking (yes or no), and alcohol consumption status (yes or no). Within each graph, taste clusters without a common letter differ (*P* < 0.05).

Finally, we compared amount consumed (g/d and percentage of energy) from the different taste clusters by participant weight status, with multivariable adjustment (**Figure 4**). Individuals in the overweight category (compared with nonoverweight) tended to consume more foods from each taste cluster, but those differences were subtle. However, those who were overweight consumed a significantly greater proportion of energy from savory–fatty tasting foods (*P* < 0.001) and less energy from sweet–fatty (*P* = 0.004) and neutral (*P* < 0.001) tasting foods, compared with their nonoverweight counterparts.

## Discussion

We investigated the association between taste intensity and nutrient content of a wide range of foods that differ in their degree of processing to understand whether taste–nutrient relationships are affected by food processing and whether different taste clusters contribute to differences in energy intakes. Our findings show a positive correlation between sweet taste and mono- and disaccharide content, between umami and protein content, between salt and sodium content, and between fat taste and fat content of foods. Dietary energy content was also positively correlated with both salt and fat tastes and weakly correlated with umami and sweet taste. Participants who had the highest average daily energy intakes or were overweight derived a significantly greater proportion of their energy from processed foods rather than UPFs, and this energy was higher from savory–fatty and lower from neutral tasting foods than those who had lower energy intakes and were normal weight. Within the population, 80% of their energy intake was dominated by savory–fatty and neutral foods compared with the other taste clusters, and this was independent of their energy intake and weight status.

Our findings are in line with previous studies from the Netherlands (29, 30), Australia (31), Malaysia (30), the United States (33), and France (32), which demonstrated that sweet taste was associated with mono- and disaccharides, salt and umami taste were both positively associated with sodium and protein content, and fat sensation was associated with fat content. Collectively, these findings suggest a robust relation between taste quality and intensity and the associated nutrient content of foods and beverages that is consistent across diverse food supplies. As shown previously, sweet taste is strongly positively correlated with sugar content but not energy content, which is significantly associated with salt, umami, and fat sensations (30, 31, 33). The energy density of many of the foods included in the current comparison is largely determined by their fat content (47), which in turn was correlated with perceived salt, umami, and fat sensation. However, when beverages were removed from the analysis, sweetness was associated with energy content in ultra-processed foods in our study.

The taste–nutrient relationships were maintained across different categories of food processing, although the strength of these relations differed across processing levels. There was a stronger association between fat taste and fat content for ultra-processed foods, reflecting higher levels of the taste substrate within this category. A slightly weaker association between sweet taste and mono- and disaccharide content was also reported in ultra-processed foods, which may be due to recent advances in reformulating sugar-containing foods with nonsaccharide sweeteners to maintain their perceived sweet intensity while supporting a reduction in overall sugar content. These results are in line with recent findings that show that discretionary foods (e.g., confectionary or snacks) provide a similar level of sensory stimulation to nonprocessed or minimally processed foods (e.g., fruit, vegetables, grains, and dairy) relative to their nutrient contents while being higher in energy content (32, 48).

Our findings demonstrate that individuals with the highest average daily energy intakes derived a significantly greater proportion of their energy from processed foods (Δ 6%) rather than UPFs (Δ 3%), and this energy was higher from savory–fatty while lower from neutral tasting foods than those with lower energy intakes. For instance, a 10% increase in the contribution of savory–fatty taste to daily energy intakes was the main difference between the lowest to the highest energy intake quartiles (1246–3762 kcal/d). This finding may be attributed to the higher proportional contribution of those high sweet–fatty foods (27%) in the ultra-processed category compared with the energy-dense savory–fatty foods that dominated the processed foods. Together, these indicate that consuming a diet with heightened savory–fatty taste offers a better explanation of population variation in energy intakes than consumption of ultra-processed foods alone.

Several plausible mechanisms have been proposed to explain the savory–fatty tasting foods contributed most to higher energy intakes. A higher perceived salty taste intensity has previously been shown to “blind” the perception of differences in fat content (17) and may make it easier to passively overconsume energy. Despite the suggestion that processed foods are hyperpalatable (49), currently there is no evidence to suggest that heightened palatability due to sweet or salty tastes makes a disproportionately larger contribution to daily energy intakes (50, 51). The current findings showed no association between hyperpalatability and ultra-processed foods consumption, as has been suggested and critiqued in the past (52), and to date, there remains no empirical evidence from clinical trials for a disproportionate contribution of specific tastes of ultra-processed foods in promoting excessive daily energy intakes. Our findings suggest fat sensation and, by association, higher energy density (47) are major contributors to higher total daily energy intakes. A high salty taste intensity may mask foods with a higher fat content, thereby making it more difficult to perceive and adjust intake when faced with high energy density (17).

Notably, there was a consistency in predominant taste pattern across the lowest to highest quartiles of energy intake. Eighty percent of a participant's dietary energy intake was dominated by foods with savory–fatty and neutral tastes. This suggests that individuals tend to consume a similar pattern of tastes on a daily basis, although those consuming the most energy tend to increase intake from savory–fatty foods and to decrease intake from neutral foods. This suggests it will be possible to reduce the energy content of the diet while still maintaining the predominant taste qualities of energy sources. This finding provides new opportunities to reformulate existing food products to reduce their energy density, to provide healthier alternatives while maintaining a similar pattern of food taste encountered in the everyday diet.

Consumption of a diet with a higher proportion of energy from processed foods but not ultra-processed foods was significantly associated with being overweight, and this energy was more from savory–fatty foods and less from sweet–fatty and neutral foods. Higher consumption of processed foods in our study was associated with higher BMI, in line with the population-based study of the United Kingdom's National Diet and Nutrition Survey that demonstrated intake with processed culinary ingredients was associated with body weight but not with ultra-processed foods (53). In addition, the taste cluster results are consistent with previous findings that consumption of high quantities of salt or higher proportion of savory–fatty foods was associated with higher body fat mass and BMI (39, 54). Individuals who were overweight consumed a similar total amount of foods as their normal-weight counterparts, suggesting that people might tend to eat a consistent weight of foods, with little adjustment in response to foods with a higher energy density.

Moving beyond the conventional nutrient intake, studying diets through the lens of taste quality and intensity could provide new insights and meaningful support for obesity interventions by taking account of an individual's taste preferences and habitual eating behavior. Previous findings have suggested that sensory properties of foods may contribute to the rate of energy intake (55), and this has been shown to vary widely across food processing categories (40). Taste intake patterns provide a new comparative approach to identify differences in the sensory qualities of diets higher or lower in energy intake and offer alternative approaches to moderate dietary composition while maintaining the sensory appeal of reformulated products. Findings from the current study can be used to identify foods and taste patterns that disproportionately contribute to daily energy intakes and offer product alternatives or reformulation opportunities while still matching the current sensory appeal of the diet. Similarly, dietary “sensory” insights could be used to convey simplified public health advice to consumers and suggest food alternatives that mitigate the impact of excess energy and nutrient intakes while keeping eating pleasure at the center of diet recommendations.

A strength of the current study was the use of objectively measured taste profiles for a representative set of foods, using best practice approaches for objective sensory evaluation (56). In addition, the current study applied taste clustering to understand food intake patterns from a large, multiethnic population-based sample of adults from Singapore. A potential limitation was the use of a self-reported validated FFQ to estimate daily energy intakes, which may be subject to selective misreporting and measurement error. Similarly, there is potential for some misclassification of certain foods using the NOVA system, due to a lack of detailed information about the actual foods consumed and their preparation methods or inconsistencies in interpretation of processing definitions from NOVA, which have continued to evolve and change since originally being published (57, 58). The cross-sectional nature of our design limits the ability to draw causal inference on the role of taste and processing as determinants of differences in energy intakes, and further longitudinal studies are required to establish the direction and causality.

## Conclusion

A food's predominant taste quality is significantly associated with the macronutrient content consumed in the Singaporean diet, and this association is sustained across different degrees of food processing. Individuals with the highest average daily energy intakes or those who were overweight derived a significantly greater proportion of their energy from processed foods rather than ultra-processed foods, and this energy was higher from savory–fatty and lower from neutral tasting foods than those with lower energy intakes and normal weight status. Within the population, 80% of their energy intake were dominated by savory–fatty and neutral foods compared with the other taste clusters, and this was independent of their energy intake and weight status.

Higher taste intensity in combination with high energy density for some processed foods can potentially promote increased energy intakes in diets high in processed food consumption. However, the predominant taste intake pattern—combination of savory–fatty and neutral foods—was consistent from low to high energy intakes, suggesting it is possible to reduce dietary energy density while maintaining the preferred taste patterns associated with dietary energy intake.

## Supplementary Material

nxab318_Supplemental_File

## Data Availability

Data described in the manuscript, code book, and analytic code will be made available upon request pending (e.g., application and approval, payment, other).

## Notes

Source of support: The MEC2 follow-up study was supported by grants from the Ministry of Health, Singapore, NUS and National University Health System, Singapore. CGF, RT, and PST were supported by the Singapore Biomedical Research Council Food Structure Engineering for Nutrition and Health (grant H18/01/a0/E11, awarded to CGF).

Author disclosures: The author reports no conflicts of interest.

RMvD is a member of the Journal's editorial board.

Supplemental Tables 1 and 2 are available from the “Supplementary data” link in the online posting of the article and from the same link in the online table of contents at https://academic.oup.com/jn/.

Abbreviations used: MEC2, Multi-Ethnic Cohort Phase 2; METs, metabolic equivalent task units; UPF, ultra-processed food.
