# Supplementary material for: Taste of Modern Diets: The Impact of Food Processing on Nutrient Sensing and Dietary Energy Intake
Source: J Nutr. 2021 Oct 12;152(1):200–10. doi: 10.1093/jn/nxab318 (PMC8754564; doi:10.1093/jn/nxab318)
Supplement: nxab318_Supplemental_File [file nxab318_supplemental_file.docx]

**Taste of Modern Diets: The Impact of Food Processing on Nutrient Sensing and Dietary Energy Intake**

**Teo et. al. Online Supplementary Material**

**Supplementary Table 1** Food items and their processing categories as defined by NOVA

| **Food** | **NOVA** | **Food** | **NOVA** |
| --- | --- | --- | --- |
| Apple | 2 | Pineapple | 2 |
| Avocado | 2 | Porridge; brown rice; plain | 2 |
| Banana | 2 | Porridge; rice; mixed white and brown; plain | 2 |
| Beans; boiled without oil | 2 | Porridge; white rice; plain | 2 |
| Beansprouts; boiled without oil | 2 | Potato; boiled without oil | 2 |
| Bivalves; e.g., oysters, cockles, mussels, and scallops | 2 | Prawns | 2 |
| Blueberries | 2 | Pumpkin; boiled without oil | 2 |
| Broccoli; boiled without oil | 2 | Raw fish/sashimi | 2 |
| Cabbage; boiled without oil | 2 | Rice; mixed white & red/brown | 2 |
| Capsicum; boiled without oil | 2 | Rice; red/brown | 2 |
| Carrot; boiled without oil | 2 | Rice; white | 2 |
| Cauliflower; boiled without oil | 2 | Seaweed | 2 |
| Celery; boiled without oil | 2 | Seeds | 2 |
| Chicken; boiled/steamed/in soup | 2 | Spinach; boiled without oil | 2 |
| Coffee; no milk | 2 | Squid; cuttlefish and molluscs | 2 |
| Corn; boiled without oil | 2 | Strawberries | 2 |
| Crab | 2 | Tea; no milk | 2 |
| Cucumber | 2 | Tomato; raw | 2 |
| Dhal; boiled without oil | 2 | Vegetables; boiled/steamed/in soup | 2 |
| Dragon fruit | 2 | Yam; boiled without oil | 2 |
| Dried fruit | 2 | Yoghurt; regular | 2 |
| Durian | 2 | Alcoholic beverages | 3 |
| Egg; boiled/half-boiled/steamed | 2 | Bean curd; boiled without oil | 3 |
| Eggplant; boiled without oil | 2 | Beef; lean and fat; roasted | 3 |
| Fish; boiled/steamed/in soup | 2 | Beef; lean; roasted | 3 |
| Fruit or veg juice; 100% juice | 2 | Begedil (potato patty); roasted without oil | 3 |
| Gourd; boiled without oil | 2 | Canned fish | 3 |
| Grape | 2 | Chapati | 3 |
| Guava | 2 | Cheese inc. cheese spread | 3 |
| Kailan; boiled without oil | 2 | Chicken curry w coconut | 3 |
| Kang kong; boiled without oil | 2 | Chicken curry w/o coconut | 3 |
| Kiwi | 2 | Chicken/turkey ham | 3 |
| Lettuce | 2 | Chicken; deep-fried | 3 |
| Lotus root; boiled without oil | 2 | Chicken; roasted/grilled/baked | 3 |
| Mango | 2 | Chicken; stewed/braised | 3 |
| Meat; boiled/steamed/in soup | 2 | Chicken; stir-fried/pan-fried | 3 |
| Melon | 2 | Coffee; low fat milk | 3 |
| Milk; low fat | 2 | Coffee; no milk; added sugar | 3 |
| Milk; non-fat | 2 | Coffee; skimmed milk | 3 |
| Milk; whole | 2 | Coffee; whole milk | 3 |
| Mushrooms; boiled without oil | 2 | Cured pork products | 3 |
| Mustard greens; boiled without oil | 2 | Dumpling | 3 |
| Nuts | 2 | Egg based mixed dishes (containing bean curd) | 3 |
| Oats | 2 | Egg based mixed dishes (containing chicken) | 3 |
| Okra; boiled without oil | 2 | Egg based mixed dishes (containing fish) | 3 |
| Orange | 2 | Egg based mixed dishes (containing pork) | 3 |
| Organ meat | 2 | Egg based mixed dishes (containing vegetables) | 3 |
| Other beans; boiled without oil | 2 | Egg; fried/scrambled/braised | 3 |
| Papaya | 2 | Fish curry w coconut | 3 |
| Pear | 2 | Fish curry w/o coconut | 3 |
| Peas; boiled without oil | 2 | Fish; deep-fried | 3 |
| **Food** | **NOVA** | **Food** | **NOVA** |
| Fish; roasted/grilled/baked | 3 | Vegetables; stewed/braised | 3 |
| Fish; stewed/braised | 3 | Vegetables; stir-fried/pan-fried | 3 |
| Fish; stir-fried/pan-fried | 3 | 3-in-1 cereal | 4 |
| Flavoured rice dish (mixed with meat/veg) | 3 | Bean curd dessert | 4 |
| Fried onion | 3 | Beef burger (incl. bread bun) | 4 |
| fried rice with vegetables | 3 | Biscuits/cookies; with filling or with chocolate | 4 |
| Glutinous rice | 3 | Bread; white | 4 |
| Idli | 3 | Bread; wholemeal | 4 |
| Meat curry w coconut | 3 | Bread; with fruit/nut/seeds | 4 |
| Meat; curry w/o coconut | 3 | Breakfast cereal; flavoured; contains wholegrains | 4 |
| Meat; deep-fried | 3 | Breakfast cereal; flavoured; not wholegrain | 4 |
| Meat; stewed/braised | 3 | Breakfast cereal; mixed; contains wholegrains | 4 |
| Meat; stir-fried/pan-fried | 3 | Breakfast cereal; mixed; not wholegrain | 4 |
| Murtabak | 3 | Breakfast cereal; plain; contains wholegrains | 4 |
| Mutton and lamb; lean and fat; roasted | 3 | Breakfast cereal; plain; not wholegrain | 4 |
| Mutton and lamb; lean; roasted | 3 | Cake | 4 |
| Noodles; dry | 3 | Canned/bottled coffee | 4 |
| Noodles; fried | 3 | Canned/bottled tea | 4 |
| Noodles; in gravy | 3 | Carbonated soft drinks; regular | 4 |
| Noodles; in soup | 3 | Carbonated soft drinks; sugar free | 4 |
| Other clear soup | 3 | Cheese; reduced fat | 4 |
| Pasta; not wholemeal | 3 | Chocolate | 4 |
| Pasta; wholemeal | 3 | Coffee; 2-in-1 | 4 |
| Pasta; with creamy sauce | 3 | Coffee; 2-in-1added sugar | 4 |
| Pasta; with meat/fish/veg | 3 | Coffee; 3-in-1 | 4 |
| Pasta; without any sauce | 3 | Coffee; 3-in-1added sugar | 4 |
| Pork; lean and fat; roasted | 3 | Coffee; condensed milk | 4 |
| Pork; lean; roasted | 3 | Coffee; condensed milk; added sugar | 4 |
| Porridge; brown rice; flavoured | 3 | Coffee; creamer; no sugar | 4 |
| Porridge; rice; mixed white and brown; flavoured | 3 | Coffee; creamer; no sugar; added sugar | 4 |
| Porridge; white rice; flavoured | 3 | Coffee; evaporated milk | 4 |
| Preserved vegetables | 3 | Coffee; evaporated milk; added sugar | 4 |
| Raita | 3 | Coffee; low fat milk; added sugar | 4 |
| Rasam | 3 | Coffee; skimmed milk; added sugar | 4 |
| Rice roll | 3 | Coffee; soya milk | 4 |
| Roti prata; plain or w egg/cheese | 3 | Coffee; soya milk; added sugar | 4 |
| Salted fish/dried fish | 3 | Coffee; whole milk; added sugar | 4 |
| Sushi roll/nigiri | 3 | Coleslaw | 4 |
| Tea; low-fat milk | 3 | Cream soup | 4 |
| Tea; no milk; added sugar | 3 | Filled buns; savoury | 4 |
| Tea; skimmed milk | 3 | Filled buns; sweet | 4 |
| Tea; whole milk | 3 | Flavoured rice | 4 |
| Tea; whole milk; added sugar | 3 | French fries | 4 |
| Thosai; plain or with filling | 3 | French toast | 4 |
| Tomato sauce (pasta) | 3 | Fried chips and crackers; savoury | 4 |
| Vegetable curry w coconut | 3 | Fruit or veg juice drink; not 100% juice (incl. cordial) | 4 |
| Vegetable curry w/o coconut | 3 | Gravy with coconut | 4 |
| Vegetable dishes (containing chicken) | 3 | Gravy without coconut | 4 |
| Vegetable dishes (containing fish) | 3 | Ice cream | 4 |
| Vegetable dishes (containing pork) | 3 | Jam; marmalade and honey | 4 |
| Vegetables; deep-fried | 3 | Kaya | 4 |
| Vegetables; roasted/grilled/baked | 3 | Lontong | 4 |
| **Food** | **NOVA** | **Food** | **NOVA** |
| Malted drink; 2-in-1 | 4 | Salad dressing; cream-based; low fat | 4 |
| Malted drink; 2-in-1added sugar | 4 | Salad dressing; cream-based; not low fat | 4 |
| Malted drink; 3-in-1 | 4 | Salad dressing; oil-based | 4 |
| Malted drink; 3-in-1added sugar | 4 | Savoury biscuits | 4 |
| Malted drink; condensed milk | 4 | Snack; sweet; deep-fried | 4 |
| Malted drink; condensed milk; added sugar | 4 | Soya milk; low sugar | 4 |
| Malted drink; creamer; added sugar | 4 | Soya milk; not low sugar | 4 |
| Malted drink; creamer; no sugar | 4 | Sports drinks | 4 |
| Malted drink; evaporated milk | 4 | Spread; hazelnut and chocolate-flavoured | 4 |
| Malted drink; evaporated milk; added sugar | 4 | Sweet biscuits; no filling | 4 |
| Malted drink; low-fat milk | 4 | Sweet desserts and kueh (with coconut) | 4 |
| Malted drink; low-fat milk; added sugar | 4 | Sweet desserts and kueh (without coconut) | 4 |
| Malted drink; no milk | 4 | Sweet desserts in soup (with coconut) | 4 |
| Malted drink; no milk; added sugar | 4 | Sweet desserts in soup (without coconut) | 4 |
| Malted drink; skimmed milk | 4 | Tea; 2-in-1 | 4 |
| Malted drink; skimmed milk; added sugar | 4 | Tea; 2-in-1added sugar | 4 |
| Malted drink; soya milk | 4 | Tea; 3-in-1 | 4 |
| Malted drink; soya milk; added sugar | 4 | Tea; 3-in-1added sugar | 4 |
| Malted drink; whole milk | 4 | Tea; condensed milk | 4 |
| Malted drink; whole milk; added sugar | 4 | Tea; condensed milk; added sugar | 4 |
| Margarine | 4 | Tea; creamer no sugar | 4 |
| Milkshake | 4 | Tea; creamer; added sugar | 4 |
| Other non-carbonated sweetened drinks | 4 | Tea; evaporated milk | 4 |
| Pancake/hotcake/waffle | 4 | Tea; evaporated milk; added sugar | 4 |
| Pastry | 4 | Tea; low-fat milk; added sugar | 4 |
| Peanut butter | 4 | Tea; skimmed milk; added sugar | 4 |
| Pizza | 4 | Tea; soya milk | 4 |
| Powdered nutrition drink | 4 | Tea; soya milk; added sugar | 4 |
| Processed chicken products | 4 | Yoghurt drink | 4 |
| Puffs and pies | 4 | Yoghurt, low fat | 4 |
| Puri | 4 |  |  |

NOVA classification; 2= unprocessed; 3= processed; 4= ultra-processed

**Supplementary Table 2** Pearson correlation between taste intensity and nutrients across unprocessed, processed, and ultra-processed food items (*N*=263)

| **Model: Foods and Beverages** | Energy | Protein | Fat | Carbohydrates | Mono and disaccharides | Dietary fiber | Sodium |
| --- | --- | --- | --- | --- | --- | --- | --- |
|  |  |  |  | *r* |  |  |  |
| **Unprocessed foods (*n*=72)** |  |  |  |  |  |  |  |
| Sweet | -0.02 | -0.19 | -0.11 | 0.30^**^ | 0.72^**a^ | 0.16 | -0.15 |
| Sour | -0.10 | -0.15 | -0.10 | 0.02 | 0.34^*a^ | -0.06 | -0.05 |
| Bitter | -0.12 | -0.02 | -0.04 | -0.20 | -0.17 | -0.03 | -0.12 |
| Umami | 0.14 | 0.48^**^ | 0.12 | -0.16 | -0.26^*^ | -0.26^*^ | 0.76^**^ |
| Salt | 0.33^*^ | 0.58^**^ | 0.29^*^ | -0.08 | -0.27^*^ | -0.03^a^ | 0.67^**^ |
| Fat sensation | 0.46^**^ | 0.59^**^ | 0.43^**a^ | -0.02 | -0.13 | 0.05 | 0.55^**^ |
|  |  |  |  |  |  |  |  |
| **Processed foods (*n*=82)** |  |  |  |  |  |  |  |
| Sweet | -0.15 | -0.10 | -0.14 | 0.22 | 0.49^**^ | -0.10 | -0.10^a^ |
| Sour | -0.03 | -0.04 | -0.02 | -0.05 | 0.01 | 0.02 | 0.16 |
| Bitter | -0.44^**^ | -0.34^**^ | -0.31^**^ | -0.23^*^ | 0.01 | -0.24^*^ | -0.37^**^ |
| Umami | 0.29^**^ | 0.50^**^ | 0.26^*^ | -0.11 | -0.07 | 0.03 | 0.51^**^ |
| Salt | 0.46^**^ | 0.33^*^ | 0.50^**^ | -0.04 | -0.14 | 0.09 | 0.55^**a^ |
| Fat sensation | 0.63^**^ | 0.45^*^ | 0.62^**^ | 0.03 | -0.05 | 0.14 | 0.58^**^ |
|  |  |  |  |  |  |  |  |
| **Ultra-processed foods (*n*=109)** |  |  |  |  |  |  |  |
| Sweet | -0.13 | -0.28^*^ | -0.04 | -0.09 | 0.42^**^ | -0.23^*^ | -0.47^**^ |
| Sour | -0.05 | -0.10 | 0.11 | -0.17 | -0.13 | -0.19 | 0.30^**^ |
| Bitter | -0.39^**^ | -0.36^**^ | -0.29^**^ | -0.39^**^ | -0.23^*^ | -0.39^**^ | -0.33^**^ |
| Umami | 0.19 | 0.46^**^ | 0.27^**^ | 0.00 | -0.24^*^ | -0.22^*^ | 0.57^**^ |
| Salt | 0.53^**^ | 0.56^**^ | 0.60^**^ | 0.27^**^ | -0.10 | 0.42^**^ | 0.84^**^ |
| Fat sensation | 0.49^**^ | 0.40^**^ | 0.74^**^ | 0.11 | 0.24^*^ | 0.19 | 0.51^**^ |
|  |  |  |  |  |  |  |  |
| **All foods combined (*N*=263)** |  |  |  |  |  |  |  |
| Sweet | -0.05 | -0.29^**^ | -0.08 | 0.18^**^ | 0.59^**^ | -0.10 | -0.32^**^ |
| Sour | -0.11 | -0.12 | -0.04 | -0.10 | 0.01 | -0.05 | 0.08 |
| Bitter | -0.31^**^ | -0.28^**^ | -0.22^**^ | -0.24^**^ | -0.09 | -0.25^**^ | -0.29^**^ |
| Umami | 0.18^**^ | 0.58^**^ | 0.25^**^ | -0.20^**^ | -0.35^**^ | -0.06 | 0.58^**^ |
| Salt | 0.41^**^ | 0.56^**^ | 0.48^**^ | -0.04 | -0.28^**^ | 0.07 | 0.70^**^ |
| Fat sensation | 0.53^**^ | 0.51^**^ | 0.65^**^ | 0.03 | 0.03 | 0.03 | 0.61^**^ |

Correlation (2-tailed) significant at ^**^*P*<0.01, ^*^*P*<0.05. Different from ^a^ultra-processed, and ^b^processed foods based on non-overlapping 95% confidence intervals
